# A Novel Method for Investigating the Role of Reflux Pattern in Color Doppler Ultrasound for Grading of Varicocele

**DOI:** 10.1038/s41598-018-24890-2

**Published:** 2018-04-25

**Authors:** Seyed Morteza Bagheri, Fatemeh Khajehasani, Hamed Iraji, Iman Fatemi

**Affiliations:** 1 Department of Radiology, Hasheminejad Kidney Center (HKC), Iran University of Medical Sciences, Tehran, Iran; 2 Department of Radiology, Kerman University of Medical Sciences, Kerman, Iran; 3 Physiology-Pharmacology Research Center, Rafsanjan University of Medical Sciences, Rafsanjan, Iran; 4 Department of Physiology and Pharmacology, School of Medicine, Rafsanjan University of Medical Sciences, Rafsanjan, Iran

## Abstract

Varicocele is the most common correctable cause of infertility. Color Doppler Ultrasound (CDUS) has a sensitivity of 97% and specificity of 94% for diagnosing this condition. This study aimed to propose a new pattern of scrotal Doppler for predicting the severity of varicocele. An observational study was conducted from January 2016 to January 2017 on 120 testes units in 60 patients. Scrotal CDUS and semen analysis were done in all participants. Patients were evaluated for reflux pattern, pampiniform venous plexus diameter, and venous reflux time. The ultrasonography parameters and semen analysis data were compared to assess the correlations between the results. The reflux pattern and vein diameters had a significant correlation. Also, a significant correlation was detected between the reflux pattern and reflux time. There was a significant correlation between the reflux pattern and two parameters of semen analysis namely sperm count and its motility. In conclusion, the reflux pattern classification suggested in this study can be used as a useful predictor of varicocele severity and sperm parameters in patients with varicocele.

## Introduction

Varicocele is the most common correctable cause of infertility. It is defined as an abnormal dilatation of the testicular veins and/or tortuosity of the pampiniform plexus in the scrotum^1^. The prevalence of varicocele is about 15% in the general male population, and this rate is about 40–50% in men presenting with infertility^2^. Despite new developments, the exact pathophysiology of varicocele is still poorly understood. However, several theories have been proposed, such as the absence or incompetence of valves in the internal spermatic veins and obstructed venous drainage^3^. It should be noted that majority of people with varicocele are still fertile^4^, but varicoceles can cause pain and alter spermatogenesis (deleterious effects on sperm count, motility, and morphology).

Varicocele can be diagnosed by palpation during the physical examination in a warm room, which is still the standard diagnostic method^5^. The specificity of this method is about 70% although many of the varicoceles are impalpable and asymptomatic. Such subclinical cases can be diagnosed only by imaging such as ultrasound. Color Doppler Ultrasound (CDUS) has a sensitivity of 97% and specificity of 94% for diagnosing varicoceles^6^. The diameter and reflux time of the largest vein in the pampiniform plexus are the most important criteria for the diagnosis of varicocele, and fortunately, CDUS can measure these indices (A venous reflux time more than 1000 ms is considered as a pathological case)^7^. The use of CDUS for the diagnosis of varicocele has been widely evaluated in many recent studies. However, the reliability of this technique to detect and determine the severity of varicocele is still controversial^8^.

Several methods have been introduced for the classification of varicoceles. However, none of these predict the severity of varicocele based on the results of semen analysis^9^. In this study, we introduce a new pattern of scrotal Doppler for predicting the severity of varicocele.

## Materials and Methods

### Ethics committee

The study was approved by the ethical review board of Iran University of Medical Sciences. All experiments were performed in accordance with the Declaration of Helsinki. All subjects gave written informed consent before inclusion in the study.

### Patient Selection

We conducted an observational study from January 2016 to January 2017 using CDUS on 120 testes units in 60 patients referred by a urologist to the department of radiology in Hasheminejad Kidney Center (HKC) to rule out the possibility of varicocele. Scrotal CDUS and semen analysis were done in all patients. Those with undescended testis, inguinal hernia, hydrocele, genitourinary trauma, infection, scrotal mass, history of scrotal surgery, and inflammation of the testis or epididymis were excluded from the study.

We introduced a new classification method for assessing the severity of the varicocele according to the scrotal CDUS study. We classified the patterns of reflux into four grades as follows: Grade 1 (retrograde), Grade 2 (augmented), Grade 3 (enhancing), and Grade 4 (stasis) (Table 1). The scrotal CDUS was done by an experienced genitourinary radiologist (with 12 years of experience) using voluson 730 ultrasonography equipment with a linear, multifrequency probe (5–10 MHz). First, the maximum intravenous diameter and venous flow of the patients were evaluated in a supine position without the Valsalva maneuver using CDUS. Then, in the supine position and following the Valsalva maneuver, the maximum venous diameter, venous flow, and the presence of reflux (duration of reflux and pattern of reflux) were investigated again. Then, the same measurements were reassessed in a standing position with and without Valsalva maneuver. We measured the most dilated vein observed in the pampiniform plexus to determine the maximum venous diameter (either in the supine or standing position; either with or without the Valsalva maneuver; either above or below the scrotum) (Table 2). For the duration of the reflux, we measured the longest venous reflux time (either in the supine or standing position; either with or without the Valsalva maneuver) (Table 2). For the pattern of reflux, the most severe condition was recorded (either in the supine or standing position; either with or without the Valsalva maneuver). For example, if we observed augmentation pattern in the supine position, but it was changed to the enhancement type in the standing position; the more severe pattern (enhancement type in this case) was recorded.

**Table 1:** The reflux patterns of varicoceles defined by the authors.

Reflux patterns	Features
Grade 1 (retrograde)	During the Valsalva maneuver, change in color is observed (from red to blue and vice versa), and Doppler spectrum shows venous flow reversal
Grade 2 (augmentation)	No color is observed during rest, but the color is observed (red or blue) during the Valsalva maneuver, and in Doppler spectrum, venous flow is observed only during the Valsalva maneuver
Grade 3 (enhancement)	A color (red or blue) is observed during rest, but the same color is enhanced during the Valsalva maneuver, and in Doppler spectrum, venous flow increases during the Valsalva maneuver
Grade 4 (stasis)	Although the vein diameter is more than 2 mm, no color is observed during rest or Valsalva maneuver, and in Doppler spectrum, no venous flow is observed despite the dilated veins (in gray-scale, the venous flow is sluggish or very slow)

**Table 2:** The diameter of the most dilated vein in the pampiniform plexus and the reflux time.

Diameter of testicular vein	Features
<2 mm	The diameter of the testicular vein is less than 2 mm
2–2.5 mm	The diameter of the testicular vein is between 2 to 2.5 mm
2.5–3 mm	The diameter of the testicular vein is between 2.5 to 3 mm
3–4 mm	The diameter of the testicular vein is between 3 to 4 mm
>4 mm	The diameter of the testicular vein is more than 4 mm
Reflux time	Features
<1 s	The venous reflux time during the Valsalva maneuver is less than 1 s
1–2 s	The venous reflux time duringthe Valsalva maneuver is between 1 to 2 s
2–3 s	The venous reflux time during the Valsalva maneuver is between 2 to 3 s
>3 s	The venous reflux time during the Valsalva maneuver is more than 3 s
Shunt type	Continuous venous reflux time is seen during the Valsalva maneuver
Stasis type	Although the vein diameter is more than 2 mm, the venous reflux is not seen during the Valsalva maneuver, and in gray-scale, the venous flow is sluggish

### Semen analysis

The semen analysis was done in the biochemistry laboratory of our university hospital with proper semen samples taken from patients after 3 days of sexual abstinence. We studied the sperm count per milliliter (in million/mL), total motility (in %), and normal morphology (in %).

### Statistical analysis

Statistical analysis was performed using SPSS 18 software. Continuous variables obtained after measurements in our study have been presented as means, S.E.M., and minimum and maximum values. We used Spearman correlation coefficient to analyze the correlation between the continuous variables. P-values < 0.05 were considered statistically significant.

## Results

**Figure 1:**
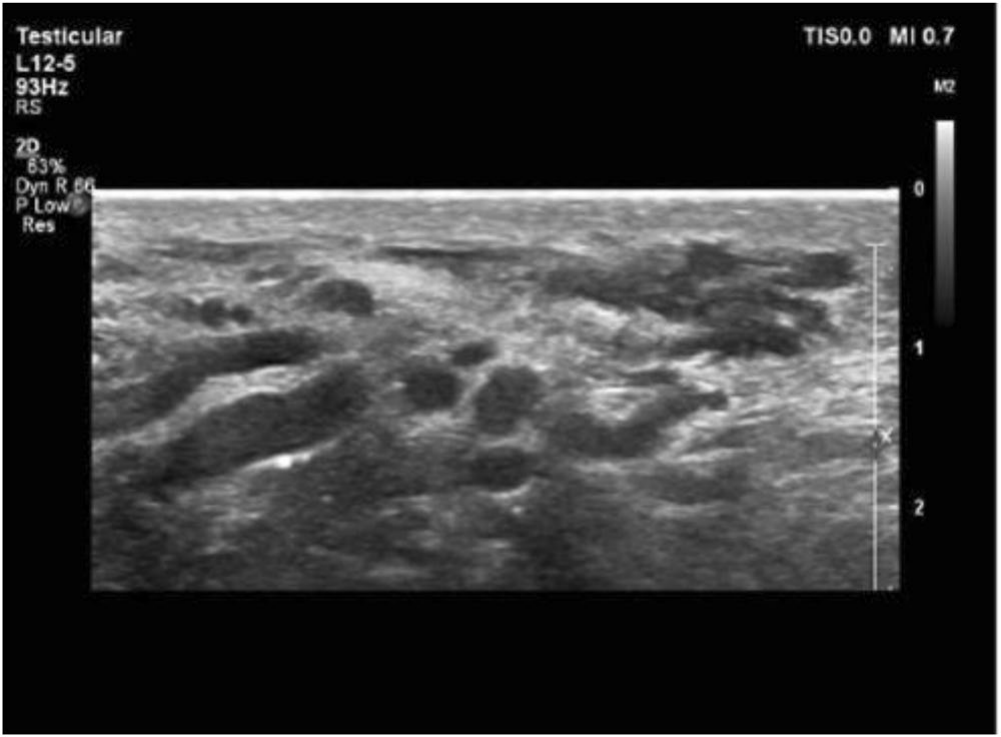
Varicocele in gray-scale and color Doppler ultrasound.

**Figure 2:**
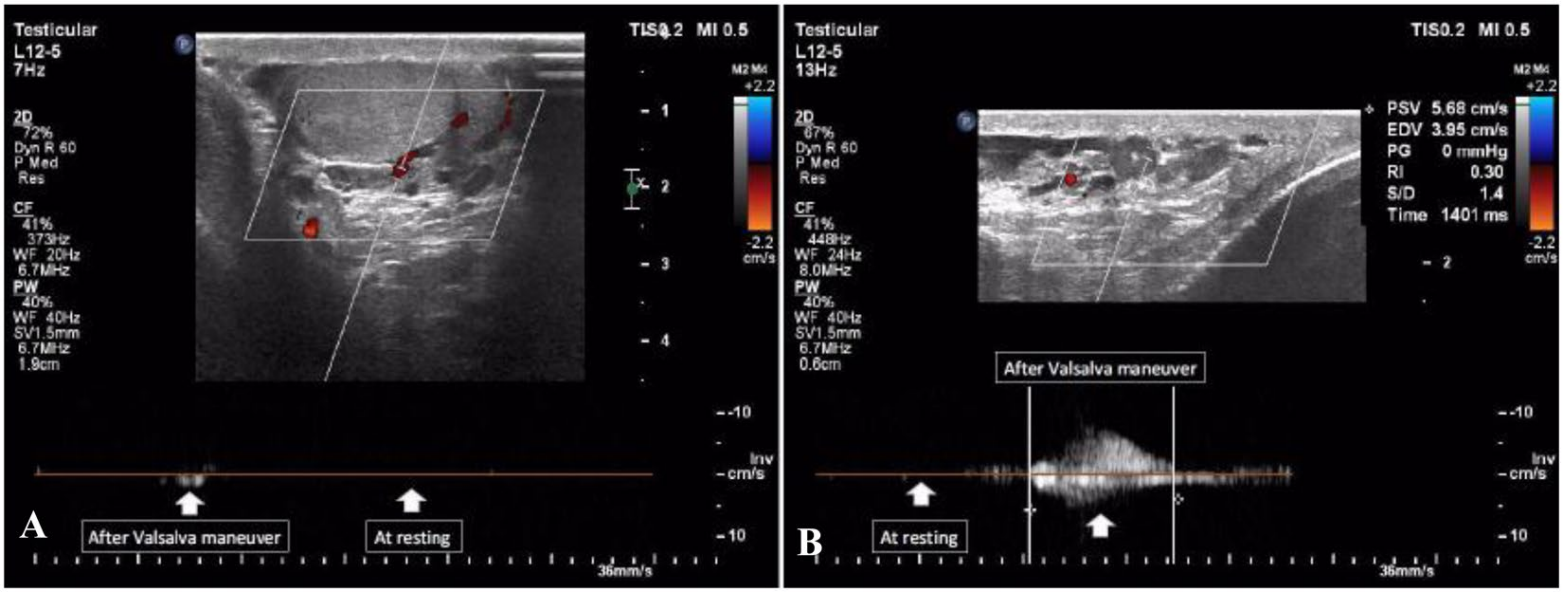
Pathologic and non-pathologic reflux. (**A**) The reflux time is less than 1000 ms and is non-pathologic; (**B**) the reflux time is about 1400 ms and is pathologic.

**Figure 3:**
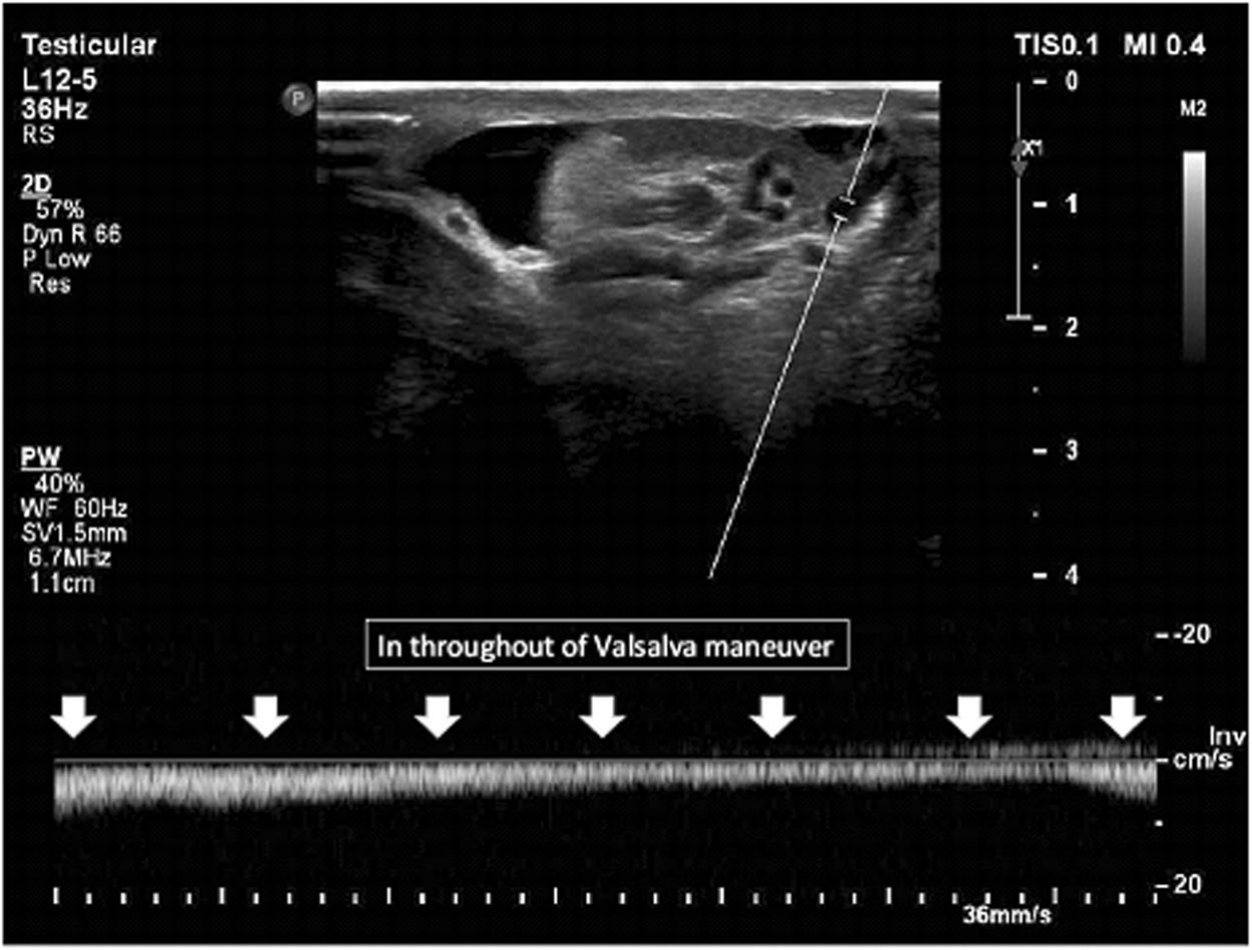
Shunt type reflux: the continuous venous reflux time seen during the Valsalva maneuver.

Figure 1 shows the varicocele in gray-scale and CDUS; Fig. 2 shows the pathologic and non-pathologic reflux time, and Fig. 3 shows the shunt-type reflux (these images have been provided for further clarification).

**Figure 4:**
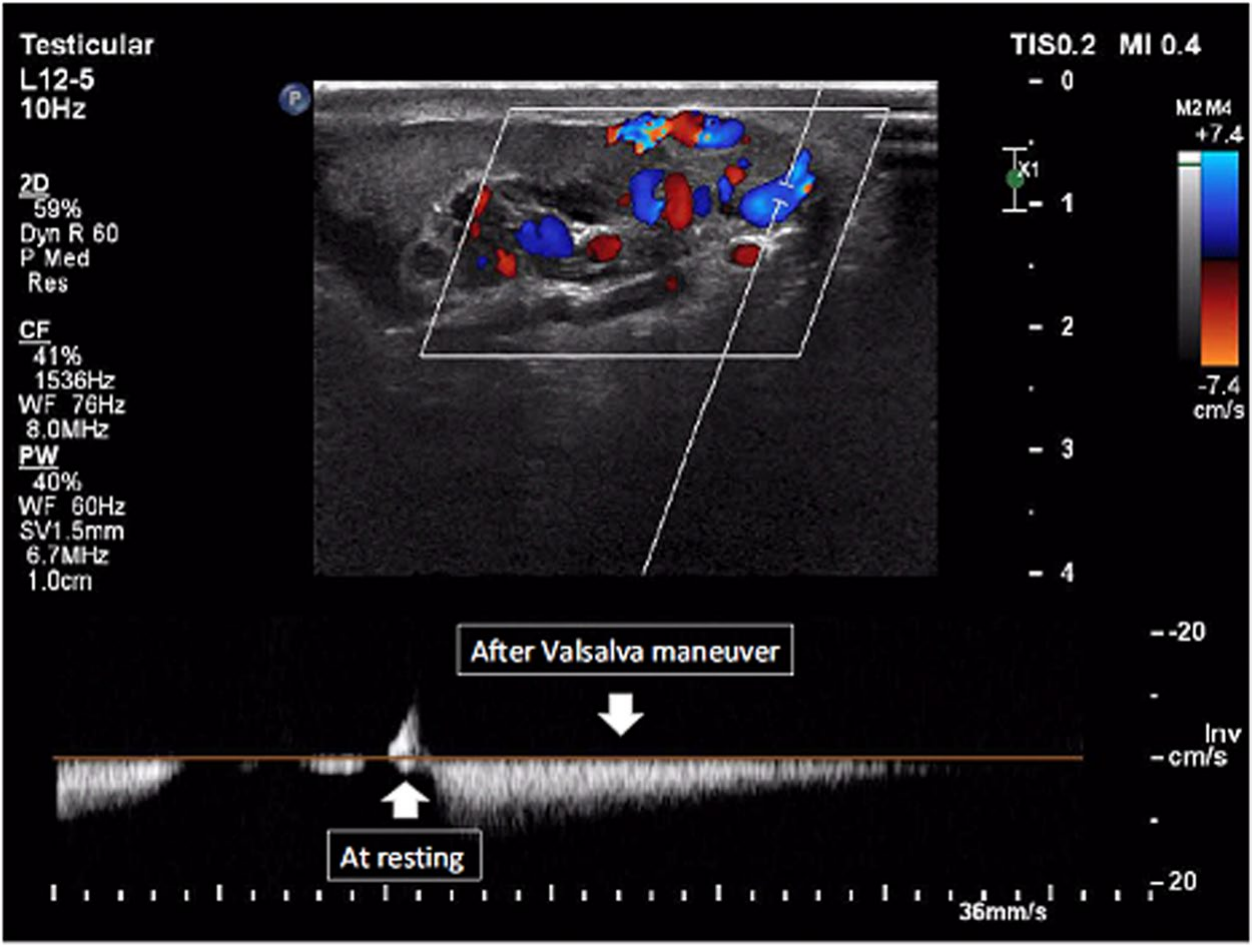
Retrograde pattern reflux: During the Valsalva maneuver, a change in color was seen (from red to blue and vice versa), and in Doppler spectrum, venous flow reversal was seen.

**Figure 5:**
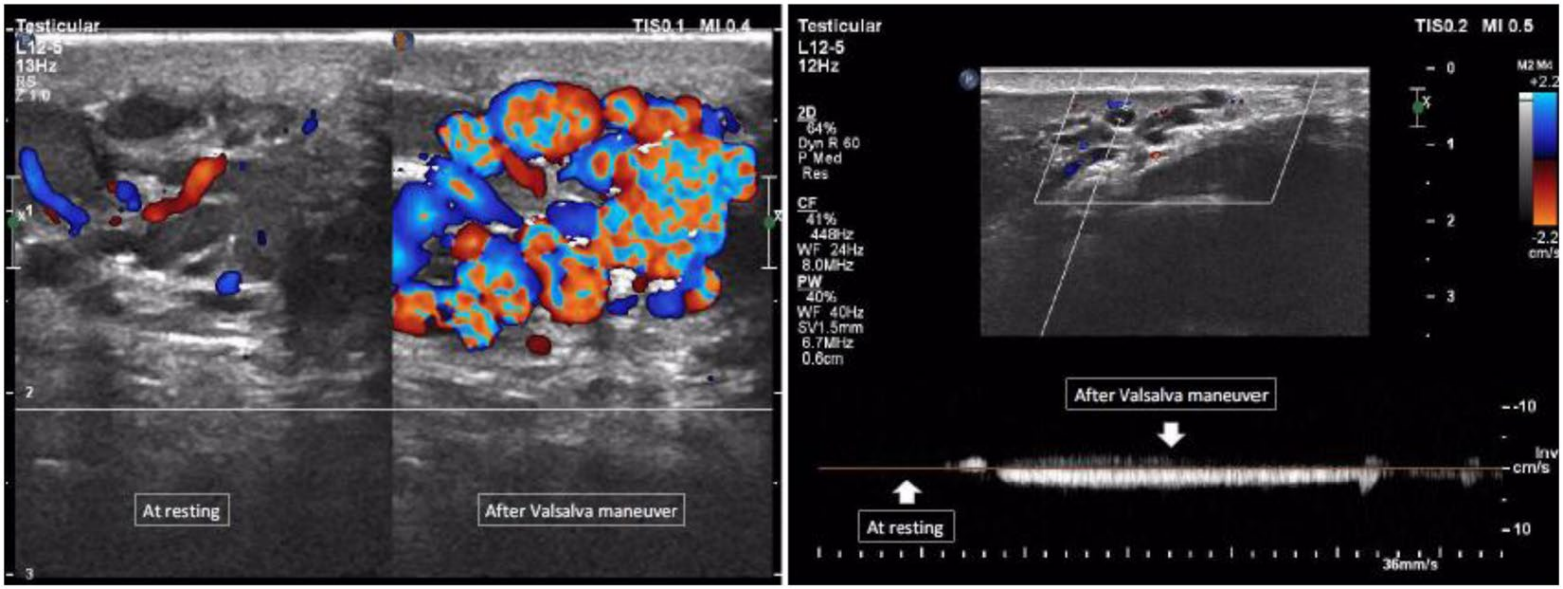
Augmentation pattern reflux: No color seen during rest, but the color was seen (red or blue) during the Valsalva maneuver, and in Doppler spectrum, venous flow was seen only during the Valsalva maneuver.

**Figure 6:**
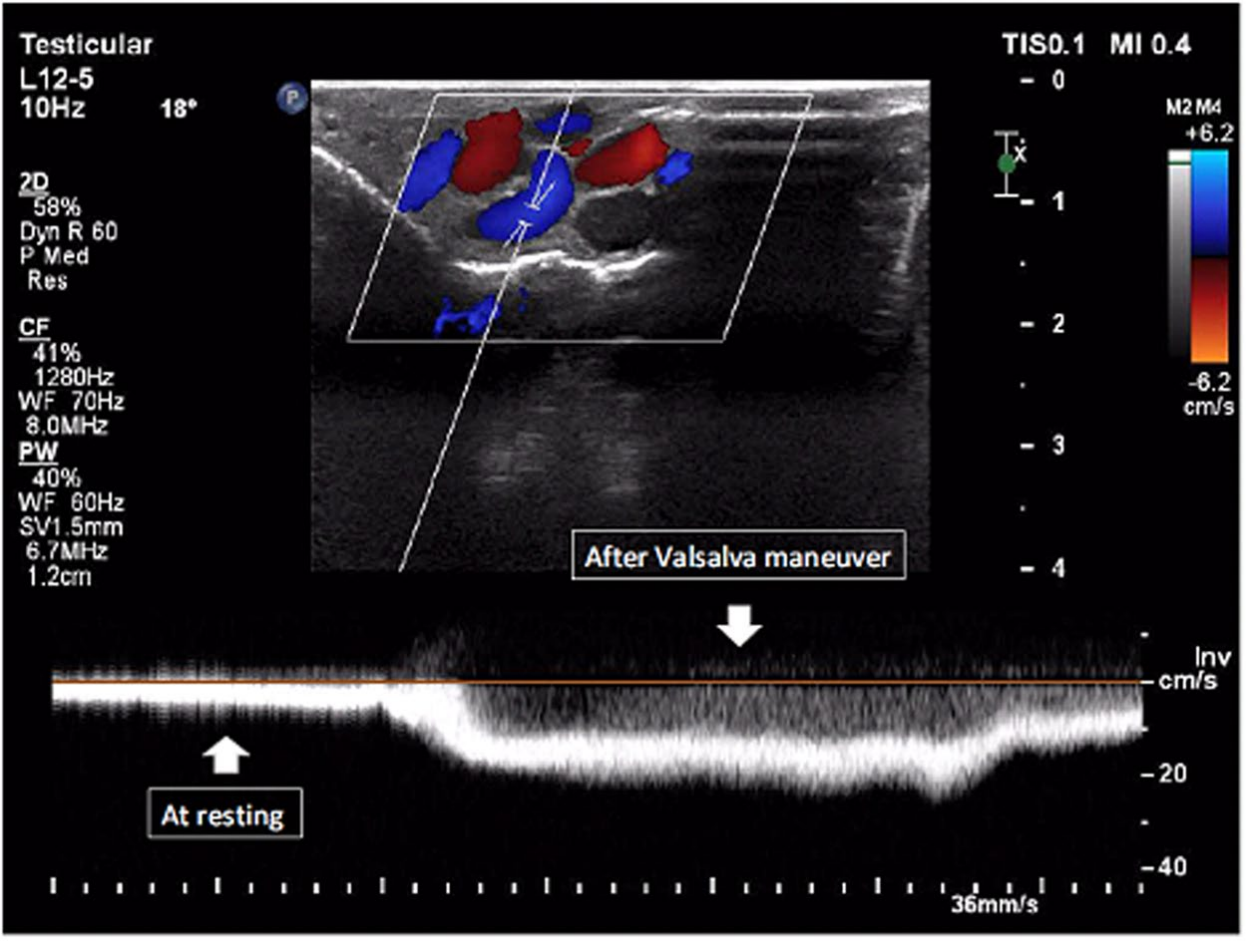
Enhancement pattern reflux: A color (red or blue) was seen during rest, but the same color enhanced during the Valsalva maneuver, and in Doppler spectrum, venous flow increased during the Valsalva maneuver.

**Figure 7:**
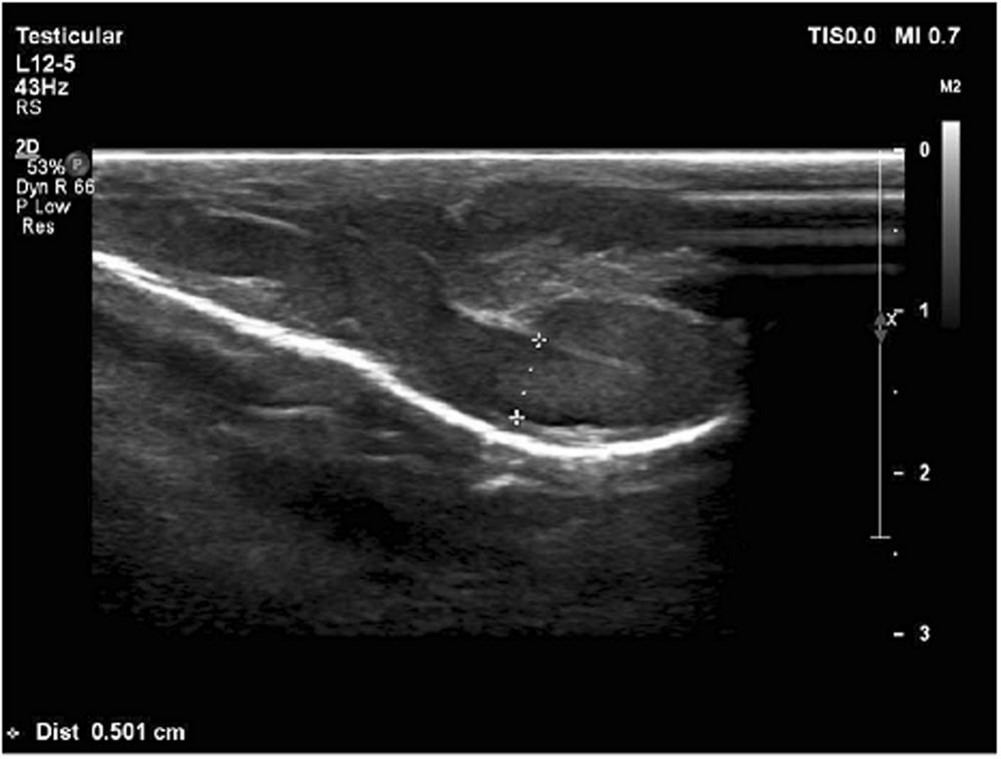
Stasis pattern: sluggish flow in gray-scale ultrasound with no color during rest or Valsalva maneuver.

This study included 60 patients; their characteristics are illustrated in Table 3. Thirty patients displayed retrograde reflux pattern (50%); the mean age was 29.5 ± 1.2 years (range: 17–47 years) (Fig. 4). Sixteen patients had augmentation reflux pattern (26.7%); their mean age was 26.6 ± 1.5 years (range: 19–35 years) (Fig. 5). Six patients displayed enhancement pattern (10%), and the mean age was 23.3 ± 1.8 years (range: 19–29 years) (Fig. 6). Finally, eight patients showed stasis pattern (13.3%), and their mean age was 31.5 ± 1.8 years (range: 24–38 years) (Fig. 7).

**Table 3:** Patient characteristics.

	Reflux pattern				
	Retrograde	Augmentation	Enhancement	Stasis	
Number of patients	30 (50%)	16 (26.7%)	6 (10%)	8 (13.3%)	
Age					p = 0.063
Range	17–47	19–35	19–29	24–38	
Mean	29.5 ± 1.2	26.6 ± 1.5	23.3 ± 1.8	31.5 ± 1.8

The correlation between the reflux pattern and the most dilated vein in the pampiniform plexus during the Valsalva maneuver is shown in Table 4. The vein diameter increased with an increase in the reflux pattern. A significant correlation was detected between the reflux patterns and vein diameters, and the correlation coefficient was 0.7 (p < 0.001). The correlation between the most dilated vein in the pampiniform plexus during the Valsalva maneuver and semen analysis parameters are shown in Table 5. A significant correlation was found between the vein diameter and sperm count (p = 0.005 and r = −0.39). Other parameters of semen analysis such as total motility and normal morphology had no significant correlation with the vein diameter.

**Table 4:** Correlation analysis between the reflux pattern and the most dilated vein in the pampiniform plexus.

		Reflux pattern				
		Retrograde	Augmentation	Enhancement	Stasis	
Vein diameter (mm)	2–2.5	16 (53.3%)	2 (12.5%)	0	0	p < 0.001
	2.5–3	8 (26.7%)	6 (37.5%)	2 (33.3%)	0	r = 0.7
	3–4	6 (20%)	8 (50%)	4 (66.7%)	2 (25%)	
	>4	0	0	0	6 (75%)	
Mean vein diameter	2.67 ± 0.14	3.38 ± 0.18	3.67 ± 0.21	4.75 ± 0.16

**Table 5:** Correlation analysis between the most dilated vein in the pampiniform plexus and sperm analysis parameters.

		Vein diameter (mm)				
		2–2.5	2.5–3	3–4	> 4	
Sperm analysis parameters	Sperm count (million/mL)	75 ± 31.57	51.16 ± 33.91	54.42 ± 23.76	32.33 ± 27.09	p = 0.005 r = −0.39
	Normal morphology (%)	36.33 ± 14.14	38.50 ± 26.62	40.20 ± 20.13	29 ± 19.24	p = 0.86 r = −0.02
	Total motility (%)	44.35 ± 13.05	43.73 ± 15.42	41.37 ± 13.27	35 ± 11.62	p = 0.176 r = −0.18

The correlation between the reflux pattern and reflux time during Valsalva maneuver is demonstrated in Table 6. A significant correlation was detected between the reflux pattern and reflux time, and the correlation coefficient was 0.71 (p < 0.001). The correlation between the reflux time and semen analysis parameters is shown in Table 7. There was no significant correlation between the reflux time and semen analysis parameters.

**Table 6:** Correlation analysis between the reflux pattern and reflux time.

		Reflux pattern				
		Retrograde	Augmentation	Enhancement	Stasis	
Reflux time (s)	1–2	4 (13.3%)	0	0	0	p < 0.001
	2–3	12 (40.0%)	2 (12.5%)	0	0	r = 0.68
	>3	4 (13.3%)	2 (12.5%)	0	0	
	Shunt type	10 (33.3%)	12 (75.0%)	6 (100.0%)	0	
	Stasis type	0	0	0	8 (100.0%)

**Table 7:** Correlation analysis between the reflux time and sperm analysis parameters.

		Reflux time (s)				Stasis type	
		1–2	2–3	> 3	Shunt type		
Sperm analysis parameters	Sperm count (million/mL)	64 ± 10.39	57.66 ± 17.04	88 ± 56.5	55 ± 28.19	32.33 ± 27.09	p = 0.125 r = −0.22
	Normal morphology (%)	36 ± 18.47	42.14 ± 22.85	28.33 ± 11.25	37.5 ± 20.76	36.75 ± 21.69	p = 0.729 r = −0.46
	Total motility (%)	46.5 ± 7.5	42 ± 17.41	36 ± 6.98	46.14 ± 12.44	31.25 ± 12.03	p = 0.417 r = −0.11

The mean values of the semen analysis parameters and its correlation with the reflux pattern are presented in Table 8. There was a significant correlation between the reflux pattern and two parameters of semen analysis. The correlation coefficients of reflux pattern with sperm count and motility of sperm were −0.49 and 0.32, respectively (all p < 0.001). Although the correlation between the reflux pattern and normal morphology of sperm was not significant, it was marginally close to a significant level (p = 0.061).

**Table 8:** Correlation analysis between the reflux pattern and sperm analysis parameters.

		Reflux pattern				
		Retrograde	Augmentation	Enhancement	Stasis	
Sperm count (million/mL)	Min	1.4	12.0	32.0	11.0	p < 0.001
	Max	143.0	105.0	88.0	192.0	r = −0.49
	Mean	60.69 ± 6.85	35.25 ± 7.34	53.66 ± 10.98	89.75 ± 24.76	
Normal morphology (%)	Min	20	20	25	7	p = 0.061
	Max	85	44	50	60	r = −0.24
	Mean	46.93 ± 3.76	30.13 ± 2.21	38.33 ± 4.59	36.75 ± 7.66	
Total motility (%)	Min	22	27	34	20	p < 0.001
	Max	64	62	84	70	r = −0.32
	Mean	44.20 ± 2.55	40.38 ± 2.77	61.00 ± 9.21	48.75 ± 7.38	

## Discussion

In this study, we introduced a new pattern of scrotal Doppler for predicting the severity of varicocele. The results of our study showed that the pattern of scrotal Doppler has a high correlation with semen analysis.

Although spermatic venography is used as the standard modality for diagnosis of varicoceles, this method has some limitations. These mainly include the exposure to ionizing radiation, invasiveness, and the need for expertise. CDUS is used as the modality of choice for evaluation of testicular varicoceles because this method has some advantages such as non-ionizing and non-invasive nature and wide availability. For these reasons, CDUS study has been preferred to diagnostic spermatic venography^10^.

Over the years, different systems have been introduced for the classification of varicoceles. One of the first classification methods was developed by Dubin and Amelar which was based solely on the clinical examination of the scrotum^9^. Another approach was introduced by Sarteschi based on a combination of CDUS study and clinical findings^9^. Iosa and Lazzarini also developed a hemodynamic classification system based on the CDUS findings^11^. In none of these methods, the correlation between the severity of varicocele and the impairment of semen analysis was investigated.

The most important venous drainage of the scrotal contents is via the pampiniform plexus veins, which mainly drains into the internal spermatic vein. But there are other secondary small veins that drain a small portion of the scrotal content venous flow. The most well-known structures are the cremasteric and the differential veins, which ultimately drain into the external and internal iliac veins, respectively. In normal conditions, despite the potential occurrence of small anastomoses between these veins and the pampiniform plexus veins, there is no significant flow between these two systems. Based on the amount of dilation of the communicating veins between the two systems (the pampiniform plexus veins on one hand and the cremasteric vein as well as the differential vein on the other hand and the presence of a valve impairment in the latter two veins), the pattern of reflux in the varicocele will be different^12,13^. In a previous study, Sigmund *et al*. characterized the hemodynamic changes in varicocele as stop-type and shunt-type patterns using venography and CDUS. This characterization is based on the venous drainage of the scrotum and communication between the two venous systems^14^. Therefore, we decided to design a complete pattern for assessing the severity of reflux based on the hemodynamic changes occurring in varicocele. Accordingly, four types of reflux patterns were defined. Grade 1 (retrograde): Due to a valve failure in the internal spermatic vein, following Valsalva maneuver, the blood flow refluxes into the pampiniform plexus veins. In this grade, we observed antegrade flow in the supine position at rest due to the lack of dilatation in the communicating veins between the two mentioned venous systems (pampiniform plexus veins vs. the cremasteric and differential veins). In the supine state, the venous pressure in the pampiniform plexus was greater than the abdominal venous pressure. However, after the Valsalva maneuver, the gradient of venous pressure was reversed, and we observed that the reflux flow stopped in a few seconds. In the CDUS study, this was determined by converting the color of the veins from blue to red or vice versa (depending on the direction of the vein and position of the probe), and in the spectral Doppler study, the blood flow was reversed (i.e., the direction of the bloodstream was reversed to the line phase). We named this type of reflux as retrograde type according to the CDUS study, (Fig. 4). Grade 2 (augmented): After prolonged disease and intensification of the reflux flow, the anastomotic veins between the two venous systems are dilated, but the valves of the cremasteric and differential veins are still intact. In general, these processes lead to increased venous compliance in the scrotal veins. In this situation, the venous pressure in the scrotum is equal with that in the abdomen at rest. But following the Valsalva maneuver, due to an increase in the abdominal venous pressure, the retrograde flow and reflux are established in the veins of the pampiniform plexus. In the CDUS study, no flow was observed at rest, but following the Valsalva maneuver, the venous flow was observed blue or red. The same finding was observed in the spectral Doppler study; at rest (without Valsalva maneuver), no evidence of venous flow was detected, but following the Valsalva maneuver, the venous flow was observed above or below the phase line. We named this type of reflux as augmented type according to the CDUS study (Fig. 5). Grade 3 (enhancing): As the duration and severity of the venous reflux increases, the cremasteric and differential veins dilate slightly, but their valves remain intact. In this situation, even at rest, the intravenous pressure in the pampiniform plexus veins is higher than that in the cremasteric and differential veins. Therefore, continuous flow from the pampiniform plexus veins to cremasteric and differential veins is established. Due to increased venous pressure gradient between these two systems during the Valsalva maneuver, this flow is enhanced. In the CDUS, we observed a blue or red venous flow, and during the Valsalva maneuver, the same color intensified. Also, in the spectral Doppler study, we found a venous flow on one side of the phase line, and following the Valsalva maneuver, the velocity of venous flow in the same direction increased. We named this type of reflux as enhancing type according to the CDUS study (Fig. 6). Grade 4 (stasis): Finally, following an increase in the severity of varicocele, the dilatation of cremasteric and differential veins and valve failure occur, leading to an increase in the venous pressure in both these venous systems which is equal to or higher than the abdominal venous pressure at rest and during Valsalva maneuver. In this situation, we could not observe a clear blood flow in the veins despite significant dilatation. On the other hand, during inspiration, the abdominal venous pressure including internal and external spermatic venous system reduced, and therefore, antegrade venous flow toward the abdomen occurred. But during the Valsalva maneuver, it is not possible to return more blood flow to the scrotum. We named this type of reflux as stasis type according to the CDUS study (Fig. 7). We found that if more than one type of reflux was observed in a patient, the most severe type was to be considered. For example, if there is an augmentation type reflux in the supine position and this type of reflux becomes an enhancement type in the standing position, the more severe type of reflux is closely associated with the patient’s findings (semen analysis). This study did not investigate the different subtypes in each reflux type (different venous diameter, reflux duration time, and location of reflux that we observed), because there are numerous subtypes, and few patients of each subtype could be included. However, this can be studied using a large number of patients.

The results of our study demonstrated that the reflux time and vein diameter had no significant correlation with semen analysis (only vein diameter had a significant correlation with sperm count). Previous studies about the correlation between different parameters of varicoceles (reflux time, vein diameter, and testes size) and spermatogenesis disorder are inconclusive. For example, Ghafoori *et al*. did not find any significant correlations of vein diameter and reflux time with semen analysis in varicocele patients^15^. In another study, Keene *et al*. found no significant relationship between testes size and spermatogenesis disorder in patients aged 12 to 17 years with varicocele^16^. On the other hand, Mahdavi *et al*. have shown that the semen analysis parameters are significantly correlated with the vein diameter and reflux time but not with the testes size^17^.

An important mechanism underlying the etiology of infertility in varicocele cases is venous stasis which leads to increased intrascrotal temperature. In these cases, impairment in the cooling effect of outgoing venous plexus leads to spermatogenesis disorder^18^. Our classification didn’t show significant correlation with vein diameter and reflux time (Tables 4 and 6). According to our results, increase in grading (severity) is associated with spermatogenesis disorder due to the venous stasis (Table 8). Only the correlation of normal morphology with the reflux pattern was not significant, but the p-value (p = 0.061) was marginally close to a significant level. As observed in this reflux pattern, when the intensity of reflux increases, the grade of reflux type increases as well. Therefore, the amount of blood stasis and blood return to the scrotum increases, which is associated with elevation of scrotal temperature and spermatogenesis disturbance (semen analysis impairment).

## Conclusions

We introduced a novel radiological grading scale (Table 1) for varicocele severity based on the reflux pattern. We conclude that this classification method could be used as a reliable indicator to predict the severity of sperm abnormality during the routine assessment of varicoceles. As noted, many patients with varicoceles have a normal semen analysis, and in some patients, despite the successful surgical treatment of varicocele, semen analysis parameters do not improve. We believe that this novel categorization of varicocele helps to determine the semen analysis parameter that is impairedsemen due to varicocele and, therefore, the patients who will benefit from surgical treatment.
